# A Qualitative Study Exploring Management of Food Intake in the United Kingdom During the Coronavirus Pandemic

**DOI:** 10.3389/fpsyg.2022.869510

**Published:** 2022-04-27

**Authors:** Tennessee Randall, Chloe Mellor, Laura L. Wilkinson

**Affiliations:** School of Psychology, Faculty of Medicine, Health and Life Science, Swansea University, Swansea, United Kingdom

**Keywords:** coronavirus pandemic, quarantine, boredom eating, home cooking, panic buying

## Abstract

The coronavirus pandemic has impacted dietary quality through increased emotional eating and extended time spent at home, as well as instances of panic buying due to uncertainty over food availability. We recruited an opportunistic sample of 40 adults living in the United Kingdom (Female = 25; Mean age = 41.9 years) (SD = 14.4) without any prior history of eating disorders. Semi-structured interviews were conducted in June 2020 and focused on the impacts of the COVID-19 lockdown on eating habits and experiences of panic buying. The data were transcribed and organized using the softwares Otter and Quirkos, respectively. Reflexive thematic analysis identified positive and negative changes to eating habits. Overall, themes highlighted that effective organization was vital to manage food purchases and consumption due to a reduced shopping frequency. However, overconsumption frequently occurred due to boredom and ease of accessing energy dense foods, which had negative implications for weight and body image. After indulging, participants attempted to revert to prior eating habits and adhere to a nutritious diet. Many also expressed the importance of having enough food to feed families, which was often reported as a reason for buying extra supplies. Understanding the long-term impacts of changes to eating habits that account for the novel coronavirus context is required to preserve health and prevent unintended changes to weight.

## Introduction

The COVID-19 global pandemic disrupted life in various ways, including employment (Blustein and Guarino, 2020), mental health (Xiong et al., 2020) and food supplies (Martin-Neuninger and Ruby, 2020). Imposed quarantine enforcements produced significant stress relating to health concerns (Mattioli et al., 2020). Of interest here were changes in nutrition and eating habits during the pandemic (Robinson et al., 2021), and the potential exacerbation of disordered eating, clinically (Sideli et al., 2021) and in the general population (Tavolacci et al., 2021). Emerging studies have reported increased emotional and binge eating, excessive consumption of energy dense foods and preoccupation with food and body image (Kriaucioniene et al., 2020; López-Moreno et al., 2020; Puhl et al., 2020; Zachary et al., 2020; Robertson et al., 2021). Such findings can be viewed in terms of coping with emotional distress and managing mental health, which will be discussed in the following sections.

In brief, numerous studies have found significant links between negative emotions (i.e., stress, worry) and the occurrence of emotional eating throughout the pandemic (Mason et al., 2020; Renzo et al., 2020; Scarmozzino and Visioli, 2020; Shen et al., 2020; Bemanian et al., 2021; Usubini et al., 2021). Furthermore, anxiety related to COVID-19 was associated with body dissatisfaction in males and females (Swami et al., 2021). Frequent occurrence of negative affect may increase risk for disordered eating through disruption to exercise routines, comparison of body image through social media and social isolation (Rodgers et al., 2020). Notably, Czepczor-Bernat et al. (2021) found that disordered eating was significantly higher for women who had high COVID-related stress, were overweight and had high body dissatisfaction, in comparison to women with a healthy body weight, no COVID-related stress and low body dissatisfaction. These findings are consistent with the homeostatic theory of obesity (Marks, 2015) which suggests weight gain is linked to various body systems through a “circle of discontent.” For instance, the theory suggests reciprocal relationships between (1) body dissatisfaction and negative affect, (2) negative affect and consumption of energy dense foods and beverages, (3) consumption of energy dense foods and overweight/obesity, and (4) overweight/obesity and negative affect.

In addition, prolonged periods at home provided ample eating opportunities, leading to more snacking between main meals (Sidor and Rzymski, 2020). Without meaningful activities or social interaction, eating may be used as an activity to counteract boredom experienced through quarantine periods (Brooks et al., 2020). One implication is that additional snacking increased overall energy intake if other meals were not adjusted accordingly and physical activity levels had altered (Maugeri et al., 2020). Furthermore, evidence suggests that changes to eating behaviours varied across individuals. This was demonstrated by Robinson et al. (2021) who found relationships between a lower diet quality and having a higher BMI, being male and less educated. Similarly, overeating during lockdown was associated with being female, having a previous psychiatric diagnosis, having a higher BMI, and experiencing poorer mental health during the lockdown period. These findings indicate the complexity of how individual differences influence food choices, and their subsequent effect on health.

Collectively, instances of emotional eating (i.e., stress, boredom) present a risk for potential weight gain (Flanagan et al., 2020; Ghosal et al., 2020; Pellegrini M. et al., 2020). A recent systematic review by Khan et al. (2022) indicated that weight gain during the pandemic was prevalent across 7.2 – 74% of participants in comparison to weight loss rates (11.1 – 32%). Notably, weight gain was prevalent in individuals with overweight or obesity. Similarly, Khubchandani et al. (2022) reported that 48% of American adults reported weight gain during the pandemic in a population study. Adding to this, psychological distress, pre-pandemic weight status, having children at home, and the time elapsed since the last weight check were all significant predictors of weight gain over the pandemic (Khubchandani et al., 2022). These studies highlight how disinhibited eating behaviours are exacerbated by quarantine regulations, and are magnified for individuals engaged in weight management. For individuals on a weight loss programme, stress attributed to the pandemic was related to having less time to focus on weight-loss behaviours and increased difficulties with maintaining healthy eating habits (Pellegrini C. A. et al., 2020). Despite the difficulties of the pandemic, some studies have reported success in weight management programmes (Binou et al., 2021; Caldwell et al., 2022). These mixed findings indicate that the occurrence of (un)successful weight management strategies are influenced by variations to the individual’s context as a consequence of the pandemic.

Indeed, some studies highlighted positive circumstances of the pandemic which influenced healthier food choices. For instance, Ramachandran and Gill (2020) found more time available meant participants could focus on eating fresh foods. Likewise, consumption of homemade meals and fruit had increased, whereas meals eaten at restaurants or fast-food establishments had decreased (Flanagan et al., 2020). Interestingly, a longitudinal study in Italy indicated that the consumption of energy dense foods and involvement in cooking had improved over the duration of the pandemic (Caso et al., 2022). Despite improvements, some acquired habits were abandoned following the relaxation of quarantine measures. Furthermore, these findings should be interpreted with caution as the sample consisted mainly of social psychology students. Therefore, students may have provided socially desirable responses that underestimated eating habits over the pandemic.

Collectively, studies have primarily reported on the deleterious effects of the pandemic on eating behaviours in both clinical and general populations. Although imposing the strictest lockdown measures was effective for reducing COVID-19 deaths (Davies et al., 2020), this created a challenging dilemma whereby understanding how individuals manage emotional adversity is necessary to prevent further engagement with maladaptive coping strategies and unintended changes to eating behaviour that are enduring beyond COVID-19 (McAtamney et al., 2021).

Another behaviour relevant to food and the pandemic was panic buying (Arafat et al., 2020), described as purchasing groceries in excessive amounts to gain control over situations that stimulate fear and uncertainty (Islam et al., 2021). Prior to COVID-19 the panic buying literature was relatively scarce, but reviewed evidence suggests threat perception is a key feature of panic buying (Yuen et al., 2020). This was reflected in recent research whereby participants with a high perceived risk of contracting coronavirus displayed significantly more intention to hoard food (Long and Khoi, 2020). Furthermore, males and females intended to stockpile when health risks were present, suggesting panic buying is not a gender-specific behaviour (Dammeyer, 2020). According to the theory of planned behaviour, it may be relevant that positive attitudes are allocated to actions which minimize threat and enhance survival during a crisis (Sharma and Sonwalkar, 2013).

Social circumstances also influenced panic buying as positive associations were found between social interactions (i.e., COVID-19 conversations) and panic buying (Yuen et al., 2020). Similarly, 54% of examined media reports during the pandemic highlighted product shortages, stimulating fears over food availability (Arafat et al., 2020). Although evidence for social influences on panic buying is limited, a field study reported observational learning influenced decision making about food (Fishman et al., 2019). At the start of the semester (i.e., higher uncertainty), students chose food stands with the longest line, indicating the use of external information (i.e., others’ food choices). One possibility is that panic buying could occur because people believe others are better informed of the situation (Yuen et al., 2020). Alternatively, normative beliefs could explain panic buying, whereby the individual evaluates the appropriateness of their intentions based on other’s evaluations of their actions (Ajzen, 1991). Due to the mixed findings, further research is required to determine whether individual perceptions or social interactions are more prominent features of panic buying. Likewise, understanding the transmission of panic buying across populations is essential to prevent future supply-side shocks (Hobbs, 2020) and unnecessary food waste (Caulfield, 2020).

Altogether, these findings have highlighted that emotional responses to the pandemic have significantly affected eating behaviours. Indeed, the pandemic influenced and changed the food environment in ways that may not have previously been experienced (e.g., restricted access to convenience foods, empty shelves in supermarkets). As previously mentioned, although changes to weight were evident in many studies, these effects were not universal. For example, percentages of participants who experienced weight gain throughout the pandemic varied from 25.6% of adult males (Reyes-Olavarría et al., 2020), 41.7% of adolescents (Allabadi et al., 2020), and 66% of adults affected by obesity/overweight with a psychiatric diagnosis (Marchitelli et al., 2020). Therefore, there is a need for studies to understand variation across individuals. The behavioural susceptibility theory of obesity (Carnell and Wardle, 2008) may be particularly useful because the theory considers how psychological (i.e., the rewarding value of food) and biological factors (i.e., responses to hunger and fullness cues) interact with the food environment (i.e., availability of energy dense foods) to influence subsequent food intake and energy balance. This model is pertinent to the pandemic because it acknowledges how people have different susceptibilities which may account for weight variations that seem to result from various changes to both the dietary environment and physical activity (e.g., closure of exercise facilities, increased time spent at home). Although the theory considers the rewarding value of food, it is also necessary to understand the role of emotion in greater detail as findings suggest that the relationship between stress and emotional eating is mediated by emotional dysregulation (Tan and Chow, 2014). Previous studies have predominantly used quantitative methods to understand such factors (Arafat et al., 2020; Ghosal et al., 2020; Long and Khoi, 2020; Zachary et al., 2020). However, these methods may not reflect a breadth of experience because they limit the opportunity for unanticipated responses from participants on the topic of overeating as a method of emotion regulation during the pandemic. Consequently, there is a need for qualitative research to provide insights that enable exploration of the contextual factors that predispose, or buffer from negative eating habits for some individuals but not others.

To summarize, the current research will address the extent to which eating habits have changed during the pandemic due to emotional eating (i.e., anxiety, stress, boredom). Also, the study will explore management of food consumption as a consequence of changes to the food environment brought about by the pandemic (e.g., supermarket shortages, food establishment closures). Finally, the study will explore panic buying that may be driven by social factors or through individual threat perception of the pandemic. This research could inform weight management interventions in terms of emotional eating as a consequence of the pandemic. Understanding the contextual factors that underpin appetitive triggers is essential to prevent further unintended weight gain and continuous reliance on maladaptive coping strategies. For instance, individuals who place high values on food as a reward or use food to soothe emotions can be encouraged to use alternative adaptive coping strategies.

## Materials and Methods

### Participants

We followed guidelines by Malterud et al. (2016) to determine the appropriate sample size. Considering the five suggested aspects of information power, it was established that the (a) aim of the study was broad, (b) sample density was sparse, (c) research was guided by a theoretical framework, (d) quality of dialog was medium, and (e) analysis was exploratory. Furthermore, we decided upon a lower and upper sample size range that would produce a dataset that reflected the richness and complexity of the issues surrounding eating behaviours and the pandemic (Braun and Clarke, 2021). Based upon this information, we estimated that approximately 35 – 40 participants would be adequate to achieve information power. The continuation of interviews was repeatedly assessed throughout the interview process. The primary researcher (TR) was able to strengthen the research dialog by reflecting on the interview process and recognizing which questions were most effective for addressing the research aims. Collectively, the author’s social media networks were used as a starting point to recruit participants, yielding a snowball sample. Compensation was not offered for participation. A 100% of participants completed the study and therefore data were analysed for all 40 interviews. Participants were informed that the research aimed to understand their management of food during the coronavirus pandemic, and the impact of lockdown on eating habits. Sampling criteria excluded participants under the age of 18, or anyone with a current or previous eating disorder diagnosis. Participants were informed that no personal details would be included in the study and responses were anonymous. Informed consent was provided through completion of an online questionnaire hosted on Qualtrics^1^. The Swansea University, School of Psychology Research Ethics Committee approved the study.

### Data Collection

As a result of 2020 lockdown restrictions, face-to-face interviewing was not possible, so the video conferencing software Zoom^2^ was used. To ensure the connections were confidential, the end-to-end encryption function in Zoom was enabled, meaning that any communication between the interviewer and participant was limited to their personal devices. Interviews took place during the pandemic (i.e., beginning of June 2020), so participants recalled current events or those that had occurred 3 months previously. As illustrated in Table 1, interviews were semi-structured and contained a mixture of open and closed questions. When designing the questionnaire, the researchers attempted to consider general aspects of the key topics discussed above and the proposed theories mentioned (i.e., the homeostatic theory of obesity, the theory of planned behaviour and the behavioural susceptibility theory of obesity) were considered. Due to the novelty of the pandemic, the development of questions also reflected pragmatic concerns that were topical at the time of interviews. For instance, growing food at home was found to promote emotional wellbeing (Ambrose et al., 2020), which could have been a protective factor against emotional eating. Therefore, a question that generally asked about whether food growing had been attempted was included. The average interview lasted 34 min, with a range between 16 and 62 min.

**TABLE 1:** Interview questions.

Interview questions
When buying food, have you changed how you would typically shop because of the pandemic? Are you attending the supermarkets more or less? Do you have to attend more than one supermarket to buy food? Are you buying food from your local shop as opposed to traveling to your local supermarket? Have you bought food in greater quantities than normal? Are you buying food for vulnerable people who can’t leave their homes due to social isolation? Do you worry about not being able to get the food that you need? Do you plan meals in advance for yourself/family? How are you feeding yourself/family? How do you feel when you see other shoppers buying in large quantities? Have you experienced any barriers that would stop you cooking meals for yourself or you family? Have you considered growing your own produce? Are you using the delivery/takeaway service that restaurants offer? Have financial circumstances impacted ability to eat? Has your typical diet changed since the pandemic or social isolation? Do you have to work from home now? If yes, has this affected you eating habits? Do you have concerns of your weight because of isolation? If yes, are you using any strategies to manage your concerns? Have your exercise routines altered due to isolation? Generally, what impact (positive/negative) has social isolation had on your nutrition and exercise?

### Procedure

Participants were informed about the study via email and completed an online consent form. One investigator (TR) conducted interviews and reminded participants of the confidentiality of their responses. Furthermore, participants were reassured that they did not have to answer any questions which might cause discomfort. The investigator began interviews by asking about demographic details, followed by questions which explored participants experiences of buying food, meal preparation and the pandemic’s impact on diet and exercise. When answering questions, participants were encouraged to apply false names to themselves or family members to maintain confidentiality. Once finished, participants were thanked and emailed a debrief form.

### Data Analysis

Interviews were recorded on Zoom and audio files were imported to Otter^3^ for data transcription. No notes were taken during interviews and participants were allocated a unique number to conceal identities. All transcripts were uploaded to the software Quirkos for data analysis^4^. Quirkos was used to organize and code transcripts. For instance, words, sentences or paragraphs were highlighted and allocated codes, represented as a bubble on the screen. The bubble became larger as more codes were added, producing an effective visual representation for codes.

Reflexive thematic analysis was utilized (Braun and Clarke, 2006) due to the novelty of the research question (given the pandemic context) and because it has theoretical flexibility, so enables greater versatility for interpretation of patterns (Clarke and Braun, 2017). One researcher (TR) completed data analysis, which is consistent with Braun and Clarke’s (2020) recommendations for qualitative research, indicating the researcher’s subjectivity does not detract from the quality of data analysis, but rather provides an interpretative reflexive account of the knowledge and experience gained from interviews.

Prior to analysis, transcripts were read multiple times to gain a consensus of participant’s experience of food consumption and shopping habits during the initial lockdown. The coding process began by identifying words, sentences and paragraphs that were related to the research question. The process was repeated for all transcripts, whereby similar codes were merged together to form sub-themes. For example, “eating impacted mood” was a code whereby participants experienced negative alterations to mood, typically after overconsumption of energy dense foods. This code was developed into the sub-theme “impact of diet on health.” Themes were developed through the interpretation and connection of sub-themes to form a meaningful narrative that answered the research question, and was supported by evidence from participants (Vasimoradi et al., 2016). Codes were reviewed several times to ensure they captured the overall essence of the sub-themes and themes they were developed under. Following Puddephatt et al. (2019), to establish rigour, an independent researcher coded a random selection of statements (10% of full dataset) based on the developed codebook. The primary researcher (TR) also completed this task. The percentage of agreed statements was determined through dividing the total number of statements by the number of agreed statements. Initial coding agreement was 71%. Follow-up discussions between researchers resolved discrepancies across codes and ensured clarity across themes and sub-themes. The researchers reassessed statements independently and reached an agreement of 95%. Following discussions, codes were updated for the remainder of the data where necessary.

As recommended by Clarke and Braun (2013), we acknowledged any assumptions or values that we hold about the topic that potentially influenced the interpretation of results, see below for a reflexivity statement.

### Reflexivity Statement

As a researcher interested in eating behaviours (TR), social media exposure and discussions with others before interviews may have influenced expectations of eating habits. Consequently, I was probing of content concerning the pandemic’s impact on weight, prompting more detailed answers. Additionally, I was exposed to empty shelves and the difficulties obtaining basic ingredients (e.g., eggs) in supermarkets. Therefore, I believed the prevalence of stockpiling would be higher and was surprised when some participants revealed no such difficulties. Consequently, I realized the concept of stockpiling is subjective and depends on individual circumstances (e.g., family’s shopping habits compared to lone adults).

Also, being a woman, other female participants may be more comfortable discussing weight concerns. Although the sample did not report a history of eating disorders, I acknowledge the discussion of eating habits could produce cautious responses, due to apprehension of evaluation. However, I remained open to responses and mitigated perceptions of judgement by acknowledging experiences from the participant’s perspective.

As the last author (LW), I provided supervision to the first author and this was likely influenced by the overarching interests of my research group which is concerned with eating behaviour and weight management, and is situated in a psychology department. Whilst I have conducted and supervised qualitative research previously, my background is predominantly quantitative. Nonetheless, I acknowledge and embrace the differences in epistemological approach, and I altered my supervisory approach accordingly, encouraging reflexivity and understanding of the co-creation of knowledge within the context of the specific study.

## Results

The convenience sample consisted of 40 participants; 25 (62.5%) were female. Participant’s mean age was 41.9 years (SD = 14.4) and were mainly living in Wales (*N* = 30). The remaining 10 participants lived in England. Table 2 contains frequencies and percentages for relationships status, employment status and living circumstances during the first lockdown. Approximately half of participants (55.3%) reported living with children during the first lockdown. Furthermore, the mean age of children was 10.8 years (SD = 4.5) and almost 75% of participants were either married or in a relationship. No participants reported being unemployed and most participants revealed they did not shield during the first lockdown (*N* = 34). Data were missing for some demographic details, but this has been highlighted in the table by the total responses.

**TABLE 2:** Demographic data of participants.

Demographic variables	Frequency	Percentage
Gender		
Female	25	62.5%
Male	15	37.5%
Total	40	100%
Location		
Wales	30	75%
England	10	25%
Total	40	100%
Age (years)	**Mean**	**SD**
	41.9	14.4
Relationship status	**Frequency**	**Percentage**
Married	15	37.5%
In a relationship	12	30%
Single	9	22.5%
Separated	1	2.5%
Widowed	1	2.5%
Missing data	2	5%
Total	40	100%
Employment status		
Working from home	11	27.5%
Furloughed	10	25%
Working away from home	7	17.5%
Other	5	12.5%
Retired	4	10%
Unable to work due to health/illness	1	2.5%
Missing data	2	5%
Total	40	100%
Were participants a parent or guardian?		
Yes	23	57.5%
No	15	37.5%
Missing data	2	5
Total	40	100%
Did participants have any children living with them during the first lockdown		
Yes	21	52.5%
No	17	42.5%
Missing data	2	5%
Total	40	100%
Age of children living with participants	**Mean**	**SD**
	10.8	4.5
Living arrangements during the first lockdown	**Frequency**	**Percentage**
Family	21	52.5%
Spouse/partner	8	20%
Alone	5	12.5%
Roommates	3	7.5%
Other	1	2.5%
Missing data	2	5%
Total	40	100%
Were participants shielding during the first lockdown		
Yes	4	10%
No	34	85%
Missing data	2	5%
Total	40	100%

### Thematic Analysis of Interview Transcripts

Data analysis produced four key themes and associated sub-themes (see Table 3) which are explained in the following paragraphs, supported by quotes from participants.

**TABLE 3:** Themes identified for management of food intake.

Theme	Sub-theme
Environmental adaptation and flexibility	Opportunity for more fresh cooking Unable to access convenience foods Organised food purchases Planning meals in advance
Dietary instability	Using food as a coping mechanism Accessibility to calorically dense foods
Eating for nutrition	Impact of diet on health Management of calorically dense foods Monitoring food intake
Perceptions of panic buying	Reduce risk to health Provide for families Social influences

### Theme 1: Environmental Adaptation and Flexibility

Most participants perceived a change to their shopping habits that followed the rules and regulations within supermarkets. Participants felt that they could not “just pop over as and when” [F, 40 years old (yo)] so adapted accordingly. Furthermore, being at home more than usual meant greater flexibility around cooking meals which is considered in the following sub-themes, (1) opportunity for more fresh cooking, (2) unable to access convenience foods, and (3) organisation of food.

#### Sub-Theme: Opportunity for More Fresh Cooking

As food was consumed “95% at home now” (M, 29 yo), perceptions of eating more freshly cooked meals were reported by most participants. Prior work schedules meant time and energy for cooking was limited. However, being furloughed from work removed the imperative time barrier.

“There’s no chance I have any motivation or strength to cook. I would rather just put a pizza in the oven or order it…But now I finish earlier… I’ve got more time now during the day to cook for myself instead” (M, 27 yo).

Similarly, participants recognized that their ready meal consumption decreased during the lockdown, as a result of having more time available for meal preparation. “the convenience food, I mean, that’s okay if you’re busy… when I was doing school runs and things like that, so you have limited time, but now I’ve got all day… and it’s cheaper” (F, 67 yo).

Some participants cooked “to pass the time” (F, 31 yo), which enabled experimentation with cooking. Likewise, many participants started baking during the pandemic, making desserts, cookies, pizza dough and bread. “I’d be making cakes, brownies, you know there’s experimenting with flapjacks… just silly little things to keep myself amused” (F, 43 yo).

#### Sub-Theme: Unable to Access Convenience Foods

The pandemic manifested a change to the food environment by removing access to takeaway restaurants and fast-food chains.

“I would just pop out with the girls and have breakfast or go out for lunch here or grab a McDonald’s when you’re hungover. But now you can’t go anywhere, you can’t buy sort of these takeaway and snacky foods” (F, 26 yo).

Despite the reopening of takeaways, a reduced use of facilities was apparent due to uncertainty over contamination. “Sort of like fear of the unknown… do they wipe their surfaces properly, are they cooking on surfaces that you know, sort of been sprayed with anti-bacterials, but you don’t know if they wash their hands” (F, 61 yo). For some, fears lessened throughout the pandemic as the situation improved. “with the COVID number having dropped in Nottingham significantly. I think we felt a bit more confident and a bit more able to take a little more risk” (M, 42 yo).

Some participants suggested that the pandemic influenced their future use of takeaway services as they are “not really missing it to be honest” (F, 40 yo). However, eating out for one participant was a significant part of their social routine which was severely disrupted by quarantine.

“If I met my friends once a week as well for coffee, we’d end up having lunch out… I’ve missed going out for a coffee and having a chat and a laugh. Yeah, life isn’t very happy in lockdown” (F, 76 yo).

This combination of factors suggested participants’ overall diet quality had improved by “having that extra bit of nutrition” (F, 31 yo) from cooking fresh at home.

#### Organised Food Purchases

Considering grocery shopping, many participants stated that they “try to limit the amount of times” (F, 46 yo) they shopped to reduce exposure risk. Some managed this by shopping online, whereas others planned meals in advance. Also, shopping lists provided structure to supermarket visits as participants organised groceries around supplies at home, ensuring they had enough food until the next designated visit. “I was more focused on what I needed to get; I took a list. You could get it instead of just dawdling through, it was more like get in get out” (F, 27 yo). Similarly, batch cooking and freezing prepared meals were common methods to enhance the longevity of foods. “I was buying like a tray of chicken breast… butterflying them, putting them in freezer bags… you can just pull them out when you need them” (M, 29 yo). In contrast, preparing meals required more flexibility due to the availability of ingredients. For example, when participants could not get specific items from the supermarkets, they often reported substituting ingredients based on the groceries they had available at home.

Turning to shopping habits, many participants initially purchased extra groceries (i.e., long-life products) as a precautionary measure due to the risk of social isolation. In particular, participants with families were concerned about their children becoming hungry if there was not enough food available. However, worries lessened over time as stock availability improved and panic buying ceased. “We’re starting to go through stocks now… it got to the point where the cupboards were overfilled… I’ve got so many children in the house and I couldn’t risk being without” (F, 44 yo).

Considering food insecurity, finances did not affect our participant’s perceived approach to eating. However, some reported shopping differently and were more mindful of expenses toward food.

“I would just say I’ve tried just be a little bit more careful with what I buy…I’ve looked at not always buying the non-essential, so I’ve cut back on alcohol, which isn’t an essential, or I’ve bought cheaper brands of things” (F, 39 yo).

### Theme 2: Dietary Instability

Despite improvements to nutrition from fresh cooking and reduced access to convenience foods, there was a common theme whereby perceived eating habits were negatively influenced during the pandemic. Primarily in the beginning of the lockdown, the overconsumption of calorically dense foods varied, lasting from a few weeks to even a few months. Dietary instability is explored through the sub-themes, (1) using food as a coping mechanism and (2), accessibility to calorically dense foods.

#### Sub-Theme: Using Food as a Coping Mechanism

Boredom eating affected many participants during the pandemic. Participants believed this habit was stimulated by prolonged periods spent at home, without any meaningful activities that usually prevented mindless eating. “whenever I’m bored, the first thing I think about doing is eating” (F, 27 yo). Consumption of energy dense foods was linked to activities such as watching TV. For example, one participant frequently perceived a loss of control over eating due to being distracted.

“I’d be watching something or on my phone and then it would be gone. I’d be like, what happened there? You don’t even realise how much you’ve eaten or that you feel full… you’re not even paying attention to the fact you’re eating” (F, 29 yo).

Additionally, some considered the comforting effect of eating. This demonstrates that specific foods were perceived to effectively alleviate anxiety, stress and frustration experienced throughout the pandemic. For instance, buying treats was often reported to boost morale within families and keep children happy. “Just for comfort for us all really… to have nice things in the house… you know when the children, if they get upset, oh come on we’ll have a little sop and we’ll have a nice biscuit” (F, 51 yo). Relating to this, participant’s overall calorie intake increased due to perceptions of eating more energy dense foods during the lockdown. Frequently mentioned foods were biscuits, chocolate and crisps. “Junk foods as well, bought a lot of pringles and biscuits and cookies, like noodles, pasta that you just put in a pot with the dried stuff, so not a great diet” (M, 21 yo).

Some participants believed their food choices had altered during the lockdown, because they developed a habit of consuming foods uncommon to their usual diet. “Crisps, and I’m not even a great lover of crisps… It’s eating for the sake of eating” (F, 71 yo).

#### Sub-Theme: Accessibility to Calorically Dense Foods

Many identified overeating at home was related to the ease of accessing food, especially as participants reported buying more calorically dense food during the pandemic. Another believed resisting tempting foods was very difficult as their workplace provided free meals and treats during the pandemic. “Going into work we were having so much free food sent to us. Cakes sent to us, chocolates, brownies. We had so much stuff you wouldn’t believe… I was eating cupcakes at half past seven in the morning” (F, 29 yo).

Alcohol intake increased for a few participants due to multiple reasons. For instance, without work responsibilities, furloughed participants felt there were more opportunities available for drinking during the week. One participant also believed drinking at home was an opportunity to socialize with friends, without the need to worry about driving.

“Quizzes or, you know, chats with friends online like, oh let’s have a drink because I wouldn’t normally if I went down the pub I’d drive… not like getting drunk every night but more, oh I’ll have a glass of gin and tonic at home, which, you know, you would never hear me say pre lockdown.” (F, 36 yo).

### Theme 3: Eating for Nutrition

The following theme considers participants’ perceived changes to eating habits which enabled them to revert to prior eating routines. After a period of indulgence during the lockdown, the novelty of frequently eating calorically dense foods diminished and participants were keen to eat nutritious foods to compensate. Two sub-themes explore this concept, (1) impact of diet on health and (2) management of calorically dense foods.

#### Sub-Theme: Impact of Diet on Health

Many participants revealed that they had gained weight during the lockdown due to reduced activity working from home and changes to diet and exercise, leading to discomfort and unhappiness with their perceived body image “I’m not as active, which means I’m not burning it off as much as I would have been. So, the weight has started coming on and I’m really uncomfortable… really feeling paranoid now so it’s horrible” (F, 43 yo).

Also, many believed the overconsumption of energy dense foods impacted mood and wellbeing, often reporting feelings of guilt and regret for their food choices. Consequently, participants perceived that a change in mentality was necessary to modify their acquired habits and revert to a “normal” routine of eating, aiming to lose the weight gained during the pandemic. Eating nutritionally was underpinned by numerous factors, such as maintaining a good body image, health concerns (i.e., infection risk from virus) and the physical improvements observed from their diet.

“If I stay healthier… God forbid something does happen. At least my body is going to be in a fit state to fight anything that I could pick up… I’m 54. You know, I’m a target age for it so, just give myself the best chance” (M, 54 yo).

#### Management of Calorically Dense Foods

Reducing the number of treats consumed, or not purchasing calorically dense foods were perceived strategies for improved nutrition. Participants believed this was necessary to reduce accessibility, as such foods could not be eaten if they were not readily available.

“I went absolutely mental and bought like twelve varieties of biscuits… I’ve never had a biscuit drawer in the fridge, and it was full to the brim… once it was gone and we ate it all I never topped it up. And I think that’s helped not having it here” (F, 27 yo).

Adding to this, participants believed monitoring food intake enabled weight loss by providing focus on consumption. Various methods were used, including calorie monitoring through food tracking applications and the consumption of lower calorie treats, which satisfied cravings without overeating.

“We’ve changed simple things like crisps, instead of buying high in fat and high in calorie crisps… we’ve got Cheetos and Quavers and they’re like 80 to 90 calories a pack… we’re having light margarine instead of having real salted butter” (F, 21 yo).

Furthermore, intake was monitored in relation to the macronutrient content of food, as beliefs were held that avoiding some foods (e.g., high carbohydrate, high fat, fried) were necessary to eat nutritiously and lose weight. Of equal importance was the emphasis placed on consumption of lean protein sources to prevent hunger.

“If I’m having chicken it would be a good quality chicken breast and it would be a lean chicken breast… I will try to avoid high fat foods… I’ll always look for the low-fat options on the labelling system” (M, 44 yo).

Besides monitoring intake, skipping meals and forms of dieting behaviours (i.e., intermittent fasting, meal replacement shakes) were believed to create a calorie deficit and decrease overall intake in some participants. For example, participants suggested that omitting lunch enabled consumption of a larger main meal or a calorically dense snack. This was stimulated by the realization of having additional calories available to allocate to other meals.

“We’re having a good breakfast now… we’re eating sort of eleven as our breakfast time. And then we’re not having nothing through the day then. And then having a proper meal for tea” (M, 41 yo).

### Theme: Perceptions of Panic Buying

Throughout the sample, panic buying was quite rare, only 17.5% of participants reported panic buying or stockpiling during the pandemic (*N* = 7). Participants revealed a variety of reasons for their behaviour. The sub-themes representative of panic buying perceptions are (1) reduce risk to health, (2) provide for families, and (3) social influences.

#### Sub-Theme: Reduce Risk to Health

Participants acknowledged that spending prolonged time in the supermarket increased their chances of contracting the virus. Therefore, they believed it was necessary to stockpile food as a precautionary measure, both to preserve their health and to be prepared if they were required to self-isolate. Furthermore, purchasing greater quantities of foods meant participants did not have to frequently attend the supermarkets. “I think the idea was buy so much food that we won’t need to go shopping again anytime soon, so we weren’t going out as regularly” (M, 21 yo).

#### Sub-Theme: Provide for Families

Many participants with children emphasised their responsibility as a parent to provide food for their families, so would stockpile to ensure there was enough to feed the family. Interestingly, some participants acknowledged their child’s preferences for specific brands, so would purchase more when given the opportunity to ensure their child’s needs were met. “we experienced a bit with [names son] waffles … our concern and this was maybe selfish but as long as he had enough waffles to get him through a couple of weeks he’d be okay” (M, 44 yo).

#### Sub-Themes: Social Influences

Additionally, general social influences were reported to be a driving factor of panic buying. Observing others buying excessively was a perceived trigger for panic buying. “I was a bit like oh gosh I’ve got hardly anything in mine, look at there’s and then I was trying to work out are they just greedy or have they got a big family” (F, 27 yo). One participant suggested “people just kind of jump on the bandwagon” (F, 27 yo) and follow the actions of others due to the situation uncertainty. Our findings suggest social influences on stockpiling were mainly caused by observing people they did not know, as only one participant reported their decision to stockpile was influenced by a known person. “Before the lockdown I had a friend call up going quick go buy everything because they’re going to close the shop, and I was like, are they? … you’ve got such a big responsibility… you can’t just sit back” (F, 44 yo).

We note that whilst few of our participants reported engaging in panic buying themselves, many of them were aware of the behaviour more generally and readily expressed an opinion on the behaviour. Many believed panic buying was selfish because “it meant that other people had to go without” (F, 61 yo). Others speculated about the role of news stories and social media had on the prevalence of panic buying “there was probably one photo circulating on social media of an empty supermarket and it probably generated hundreds of people going well I need to go and bulk buy” (M, 41 yo). On the other hand, many tried not to impose judgement as they assessed the context as to why people might have been buying more.

## Discussion

The aim of this research was to explore how eating habits were influenced during the first lockdown of the pandemic with a particular focus on emotional eating and panic buying. Since the coronavirus pandemic this is one of the few qualitative studies (Filimonau et al., 2021; Menon et al., 2022; Razi and Nasiri, 2022) that has aimed to understand how people without diagnosed eating disorders have managed food intake in novel circumstances. Our results provide an insight into the use of food as a coping mechanism during novel circumstances. Furthermore, the findings highlight how modifications to the immediate food environment can both facilitate and ameliorate emotional eating. Using thematic analysis, four broad themes and sub-themes were established. There was a consensus that being more organised and planning meals ahead was necessary to limit supermarket visits. Altered work schedules provided the luxury of time to focus more on cooking fresh meals. However, surplus time in combination with being at home created a high susceptibility to boredom eating, leading to a lowered mood and weight gain for some. Consequently, many participants tried to eat nutritiously after a period of overindulgence and utilized strategies to manage their consumption of energy dense foods at home. Perceptions of panic buying revealed that the media accentuated the lack of food available. Also, the decision to purchase extra food supplies was influenced by the behaviour of others and individual circumstances (i.e., people with big families, buying for vulnerable people).

A key theme highlighted the opportunity to cook more fresh food at home. The circumstances of the pandemic facilitated fresh cooking by removing imposing work schedules. Consequently, more time for cooking meant participant’s perceived diet had improved by eating more fresh food. The current findings support previous research investigating the perceived barriers and facilitators to cooking (Lavelle et al., 2016). Time was also a perceived barrier as participants often relied on convenience foods due to work pressures, suggesting limited time for cooking. However, lockdown measures restricted access to takeaways and provided flexibility around meal preparation as participants were furloughed or working from home. Interestingly, despite continued access to ready meals in the supermarkets, many had reduced their ready meal consumption during the pandemic. The findings suggest the opportunities for cooking fresh food are heavily influenced by time management. In addition, Lavelle et al. (2016) reported intentions toward home cooking were facilitated by planning and organizing meals prior to food purchases. Our participants also reported eating nutritiously due to meal planning. In contrast, eating freshly cooked meals was not the main objective for organisation. Rather, considering the context of the pandemic, participants regarded planning as necessary to reduce shopping, as frequently attending supermarkets increased exposure risk to the virus. Furthermore, the prevalence of panic buying meant planning meals was often hindered by availability of ingredients in the supermarket. Consequently, participants adopted a flexible approach to cooking and adapted meals accordingly.

Despite a perceived improvement to diet, lockdown measures meant prolonged periods were spent at home and changes to work commitments meant surplus time was available. Perceptions were held by participants that these environmental changes led to the development of maladaptive eating behaviours. The occurrence of such behaviours can be interpreted through the theory of emotion regulation (Gross, 1998). The theory differentiates between two aspects of emotion regulation. Antecedent emotion regulation focuses on responding to the emotion before it has occurred to lessen its impact when the emotion takes place. For example, the uncertainty of the situation in combination with fears of contracting the virus may have led individuals to buy more food than typically needed to avoid needing to leave their home more than necessary (i.e., situation selection and modification). Also, more free time may have increased thoughts about deciding what meals to have and ensuring there was enough snacks for families (i.e., attentional deployment). Adding to this, the decision to purchase more energy dense foods might be influenced by the expectation that such foods will please family members and improve mood (i.e., cognitive change) due to the restrictions imposed. On the other hand, response focused emotion regulation which explains attempts to intensify, diminish, extend or suppress the emotion after it has been experienced. For instance, the removal of work or meaningful hobbies induced an enduring state of boredom, whereby the abundance of food in the immediate environment may have been used to suppress boredom. Studies have consistently shown that the use of suppression for regulating emotions often exacerbates emotional eating (Evers et al., 2010; Romano et al., 2021). Furthermore, a recent study by Buckland et al. (2021) found increased consumption of energy dense foods was positively associated to having higher scores on food responsiveness and emotional overeating and lower scores on emotional undereating. These findings were also evident in the current sample whereby overconsumption of energy dense foods was facilitated by the availability of foods and to regulate negative emotions. The study also found that acceptance significantly reduced the effect of having low control over cravings and consumption for energy dense snacks. However, the current sample demonstrated forms of active coping to manage energy dense food consumption by reducing purchases of energy dense foods and monitoring food intake. The current findings have implications for the management of emotional eating during lockdowns. For instance, education which focuses on how food influences mood and emotions may help individuals to make more balanced choices. Also, clinicians could devise both antecedent (i.e., having less energy dense food at home, attributing more positive values to the consumption of healthier foods) and response focused (i.e., going for a walk when feeling bored, call a friend or family member) to increase self-efficacy in individuals that they can effectively deal with negative emotions.

Of importance is the perceived engagement in disordered eating behaviours during the first lockdown. Although participants did not report having any prior eating disorders, many experienced heightened body dissatisfaction, binge eating caloric food and loss of control over eating. This is consistent with findings from the general population (i.e., no diagnosed eating disorder) in Australia, where rates of binge eating increased for 35% of participants (Phillipou et al., 2020). Moreover, the pandemic exacerbated effects for individuals with existing eating disorders. For instance, reports from the Netherlands (57%) and United States (58%) revealed concerns that spending extended time at home (i.e., a triggering environment) would aggravate eating disorders (Termorshuzien et al., 2020). There was much variability across participants concerning their perceived duration of maladaptive eating habits which ranged from weeks to months, although some did not experience any significant changes. In line with Konttinen (2020), the occurrence of overconsumption could be attributed to the continuous stress induced by the pandemic (i.e., concerns over personal safety and fears of contracting the virus), the abundance of energy dense foods in the immediate environment and genetic factors that could predispose eating behaviours. Adding to this, the initiation of dieting plans during the pandemic is also perceived to be potentially harmful. People who adopted such behaviours were almost 10 times more likely to have eating disorder symptoms than those who didn’t make any dietary changes during the pandemic (Chan and Chiu, 2021). Comparably, participants in the current study also employed various strategies to manage consumption of caloric foods. Unique to the pandemic, reduced access to convenience foods and restaurants meant shifting focus to aspects of the home environment and shopping habits. For instance, purposely not buying caloric foods was also reported by Gatzemeier et al. (2019) to reduce accessibility and manage consumption. Strategy uses could be explained by implementation intentions (Gollwitzer, 1999). This concept is a self-regulatory process which facilitates goal attainment (i.e., trying to eat nutritiously) by identifying maladaptive eating habits (i.e., boredom) and planning an alternative behavioural response that is congruent with nutrition goals. Current findings suggest many participants implemented their desired eating intentions by replacing energy dense snacks with lower calorie alternatives (e.g., fruit, yoghurts). Therefore, participants perceived their diet to be more balanced, as they did not completely deprive themselves of treats. The adoption of such strategies could protect against disordered eating by adopting dietary practices which are deemed manageable by the individual and do not exclude particular foods (King et al., 2020).

These findings highlight the complex interconnection between weight management and disordered eating habits, whereby the use of appropriate coping strategies is critical to ensure that initial eating problems do not surpass clinical thresholds. Additionally, extended waiting times have implications for service users, whereby increased distress through could result in a longer recovery time (Austin et al., 2020). Likewise, the inundation of eating disorder services could lead to increased pressure on clinicians to address backlash caused by the pandemic. Consequently, there is need for more funding for roles that can support clinicians, as the replacement of traditional face-to-face treatments with online methods has received mixed responses with regards to satisfaction and engagement with treatment (Stewart et al., 2021). Furthermore, Hamilton et al. (2020) highlighted weaknesses of the modern food system (e.g., overreliance on technology, homogenous crops) whereby future supply shocks are likely to occur, consequent of another pandemic or the emerging climate emergency. Mitigating the effects of a food system shock is essential to protect vulnerable individuals from using maladaptive forms of eating to cope with negative emotions.

Turning to food purchases, participant’s behaviour was consistent with research regarding the psychological causes of panic buying. Yuen et al. (2020) indicated the perceived threat of the situation was the most predominant cause of panic buying. In relation to the pandemic, some participants were motivated to bulk buy to prevent leaving home, as supermarkets were perceived as a high-risk environment. Also, Yuen et al. (2020) acknowledged the importance of social factors on panic buying (i.e., normative influences, observational learning) but did not consider the individual context that predisposes panic buying. For instance, many of our participants reported buying extra, indicating a responsibility to provide food for families. Although most of our sample did not report engaging in panic buying, this is consistent with Bentall et al. (2021) where over-purchasing was also minimal in British adults. The study also reported that panic buying was predicted by having a higher household income, presence of children at home, psychological distress, and increased threat sensitivity. However, the current findings contradict a recent review by Rajkumar and Arafat (2021) which indicated that higher income was a larger risk factor for panic buying. However, it seemed that as the current sample did not experience significant difficulties with obtaining food, there was no justification to purchase more than necessary.

Relating to this, one issue with food that was notably absent from the current sample was food insecurity. This could be attributed to the lack of cultural diversity amongst the current sample, as there was no representation amongst Black, African, Caribbean, or Asian populations. A recent study found that the odds of being food insecure were two times higher in ethnicities which were Mixed or White other, in comparison to White British (Yau et al., 2020). Such disparities in the experience of food insecurity across ethnic groups are also apparent in other cultures (Rezazadeh et al., 2016; Mishra and Rampal, 2020; Bukari et al., 2021; Tefera et al., 2022). Food insecurity influences a plethora of issues relating diet quality (Velde et al., 2020), eating disorder pathology (Hazzard et al., 2020), mental illness (Elgar et al., 2021), and obesity (Wu et al., 2019). Relating to the current study, it could be suggested that choice of coping mechanisms is also applicable to people with food insecurity. For instance, a study by Keenan et al. (2020) found that household food insecurity was indirectly associated to having a higher body weight through experience of distress and eating to cope. However, its notable that coping mechanisms are likely to vary depending on the severity of food insecurity and culture. For instance, food insecure individuals from Western Africa reported that cooking and sharing meals with other families was able to improve mental health (Myers et al., 2019). Likewise, Aboriginal and Torres Strait Islanders relied on extended family members for support, but the use of social comparison (e.g., others are in a worse situation) helped families to remain positive (McCarthy et al., 2018). Finally, findings from a review conducted in Malaysia reported various forms of emotional coping such as aspirations (i.e., faith that good fortune will triumph), resignation, distraction, and frustration (Sulaiman et al., 2021). Altogether, these findings highlight the importance of cultural values when considering the role of coping with distress in relation to food insecurity, overconsumption and panic buying behaviours.

The limitations of this research are attributed primarily to the sample demographics. As previously mentioned, all participants were employed or received furlough money throughout the pandemic. As participants’ ability to eat and obtain food was not significantly affected by finances, we were unable to explore food insecurity. This suggests the current findings are not generalizable to individuals who were financially constrained during the pandemic. Furthermore, the study recruited a convenience sample where the ethnicity of participants was White. Therefore, this study does not account for differences that may be apparent for people from diverse cultures. Finally, findings were based on participants with a large age range. Therefore, we cannot assume participants of all ages were equally susceptible to their perceived eating habits, and whether reported strategies were similarly effective for reducing the consumption of energy dense food.

The current findings highlight a number of opportunities for future research; firstly, there is a need to understand the long-term consequences of altered eating behaviours consequent of the pandemic, for people without a previous eating disorder. Despite participants’ perceived management of energy dense foods, its unknown whether the reported management strategies are successful when challenged with previously formed habits (i.e., eating whilst watching TV), especially in circumstances of prolonged isolation and reduced access to social support (Rodgers et al., 2020). In addition, conducting this research in food insecure populations can explore the differential challenges encountered when accessing food and the implications this has for malnutrition in families and their subsequent risks for developing an eating disorder. Finally, stockpiling throughout the pandemic disrupted the food system, as a consequence of the critical discrepancies between consumer demands and the retailer’s ability to provide supplies (Panzone et al., 2021). Considering the economic impact of stockpiling is necessary to assess the resilience of the food supply chain. However, future research should integrate an economic and a psychological understanding to better predict consumer behaviour and prevent future problems with panic buying.

## Data Availability Statement

The raw data supporting the conclusions of this article will be made available by the authors, without undue reservation.

## Ethics Statement

The studies involving human participants were reviewed and approved by the Swansea University Research Ethics Committee. The patients/participants provided their written informed consent to participate in this study.

## Author Contributions

TR and LW developed the initial idea. TR planned the study, carried out interviews, analysed the data, and wrote the original draft of the manuscript. LW supervised the research. CM assisted with researcher triangulation of the thematic analysis. All authors reviewed and edited the manuscript.

## Conflict of Interest

The authors declare that the research was conducted in the absence of any commercial or financial relationships that could be construed as a potential conflict of interest.

## Publisher’s Note

All claims expressed in this article are solely those of the authors and do not necessarily represent those of their affiliated organizations, or those of the publisher, the editors and the reviewers. Any product that may be evaluated in this article, or claim that may be made by its manufacturer, is not guaranteed or endorsed by the publisher.

## Footnotes

1
https://www.qualtrics.com/

2
https://zoom.us/

3
https://otter.ai/

4
https://www.quirkos.com/index.html
